# CD63, a new therapeutical candidate for cholesterol homeostasis regulation through extracellular vesicles?

**DOI:** 10.20517/evcna.2024.92

**Published:** 2025-03-19

**Authors:** Julien Saint-Pol, Laurence Fenart

**Affiliations:** Université d’Artois, Blood-Brain Barrier laboratory (LBHE - UR2465), Faculté des Sciences Jean Perrin, Lens F-62300, France.

**Keywords:** Extracellular vesicles, exosomes, CD63, cholesterol balance, endosomes

## Abstract

CD63 is a tetraspanin initially associated with late endosomes and contributes to numerous functions at the cell level, such as intracellular endosomal and lysosomal trafficking, adhesion, and motility. CD63 also plays a key role in the biogenesis and release of exosomes, i.e., small extracellular vesicles (EVs) of endosomal origin, facilitating the formation of multivesicular bodies (MVBs), the coordination with the endosomal sorting complexes required for transport (ESCRT) machinery, the selection of cargoes carried by future exosomes, and the fusion of MVBs with the plasma membrane for exosome release. In a recent publication in *Nature Cell Biology*, Guillaume van Niel’s team provides arguments in favor of another EV-linked function for CD63, namely the regulation of cholesterol storage and release by small EVs of endogenous origin. Complemented by two other publications from the teams of Keisuke Ito and Xabier Ostreikoetxea, which respectively describe the role of (i) mitochondrial metabolism on CD63 function and (ii) the link between the reduced CD63^+^ small EVs and dyslipidemia, these arguments highlight the key role of CD63 in the regulation of cholesterol homeostasis through exosomes and more widely small EVs in physiological and pathological conditions. Future research on CD63 may thus redefine our approach to cellular lipid management and therapeutic lipid delivery.

## CORE OF THE COMMENTARY

The tetraspanin (Tspan) family is composed of 37 small proteins in humans and characterized by (i) four spans referring to their four transmembrane domains; (ii) a small extracellular loop; and (iii) a large extracellular loop holding a specific CCG motif and between 4 to 8 cysteine residues^[1-2]^. Tspans have long been recognized for their influence on membrane and endosomal dynamics, cellular metabolism, and EV formation and function^[1,3,4]^. However, the specific mechanisms governing lipid organization and trafficking through vesicles remain under investigation^[5,6]^. By positioning CD63 - a C6-CC Tspan according to the updated Tspan classification^[7]^ - as a mediator of cholesterol distribution, Palmulli and colleagues build on foundational work that established the importance of Tspans in vesicular transport and lipid raft assembly^[8]^. CD63, previously characterized for its role in protein clustering and vesicular trafficking^[9]^, now emerges as a crucial regulator of cholesterol-rich microdomains, potentially impacting membrane integrity and signaling pathways.

**Figure 1:**
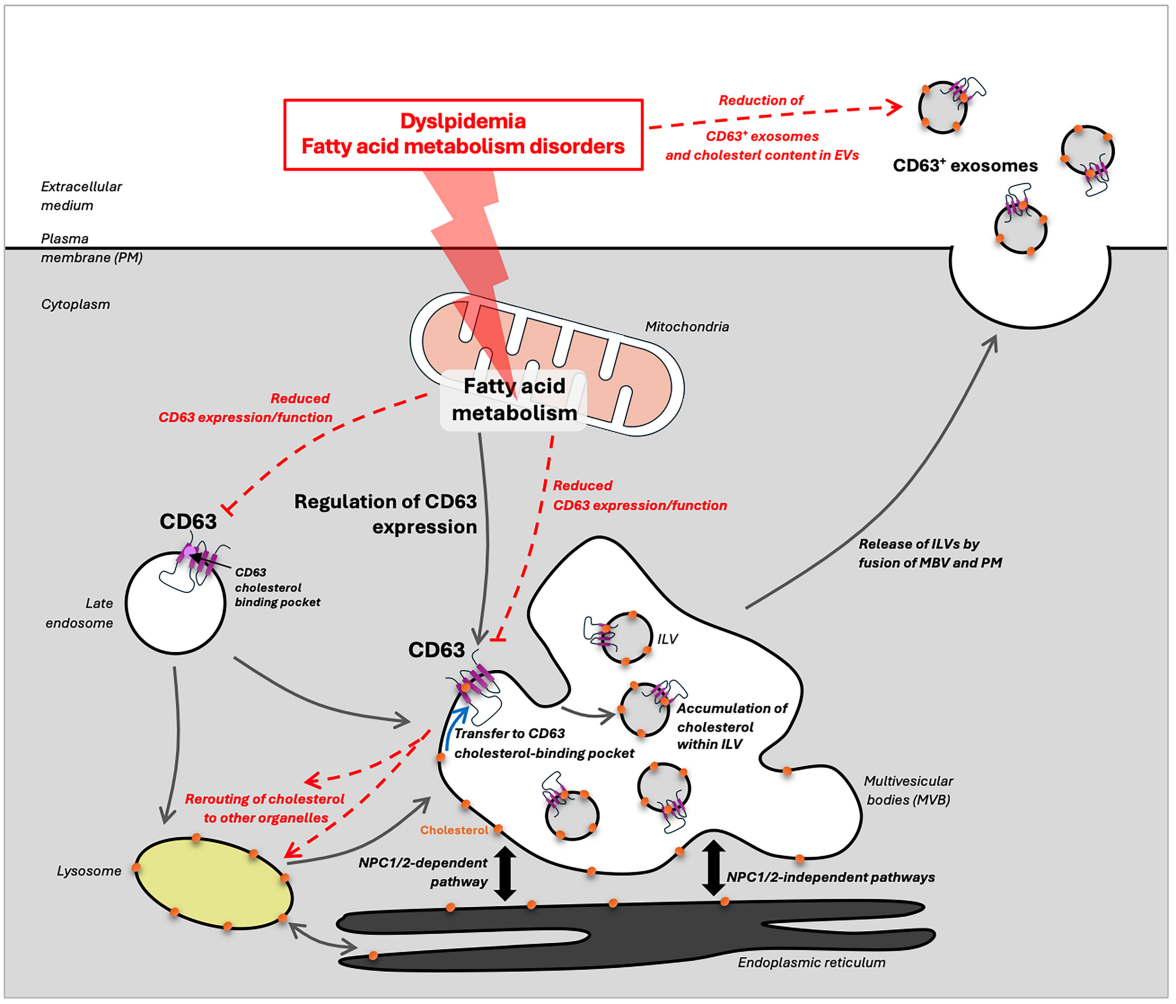
CD63 regulates the cellular cholesterol balance and export through exosomes. The expression and function of CD63, which seem to be partly regulated by mitochondrial fatty acid metabolism, promote cholesterol transfer from the membrane of the MVBs to CD63 cholesterol-binding pocket. After budding and formation of ILV within the MVB, CD63 enriches ILV in cholesterol, constituting lipid raft-like domains. The subsequent secreted CD63+ exosomes enriched in cholesterol can, therefore, deliver their lipid content to receipt cells and favor the exchanges of cholesterol with cells/organs of interest(s) through exosomes. In pathological contexts linked to lipid disorders such as fatty acid metabolism dysregulation or dyslipidemia, CD63 expression and/or function is altered, the cholesterol is rerouted to other organelles, and CD63-deficient exosomes released are poorly concentrated in cholesterol. ILV: Intraluminal vesicles; MVBs: multivesicular bodies.

Using lipidomic profiling and fluorescence microscopy, Palmulli and colleagues showed that cells expressing CD63 had higher cholesterol levels within intraluminal vesicles (ILVs), indicating that CD63 directly mediates cholesterol trafficking into these vesicles^[10]^. The deficiency of CD63 altered the intracellular cholesterol trafficking routes, rerouting cholesterol away from MVBs and toward other cellular destinations. This finding highlights CD63 as a central regulator of endosomal cholesterol balance and suggests that cells rely on CD63 to maintain lipid homeostasis within specific organelles. They also demonstrated that CD63+ extracellular vesicles (EVs) contained significantly higher cholesterol levels than EVs from CD63-deficient cells. This indicates that CD63 is critical for generating cholesterol-rich EVs, which can then carry cholesterol to recipient cells, suggesting a broader role for CD63 in intercellular lipid transfer. By structural analogy with CD81 and its capacity to bind cholesterol^[11]^, they also reveal the presence of a cholesterol-binding pocket represented by a cone-like structure and an intramembrane cavity on CD63 structure. This feature is linked to the glutamate residue in position 217 (E217) in the CD63 amino acid sequence that is localized in its fourth transmembrane domain. This newly identified structure facilitates cholesterol partitioning into the membranes of MVBs^[10]^. This pocket is essential for the precise sorting of cholesterol into ILVs within MVBs, effectively enriching ILVs and further exosomes with cholesterol. By shifting cholesterol distribution within cells, CD63 influences not only lipid storage but also the potential signaling pathways that depend on cholesterol-rich microdomains. The study suggests that CD63-mediated cholesterol sorting could have downstream effects on cell signaling, as cholesterol-rich EVs could interact with recipient cells and modulate lipid-based signaling pathways, thereby impacting cellular communication^[10]^. Based on these findings, CD63 could be involved in endolysosomal cholesterol egress pathways [Figure 1] either (i) linked to Niemann-Pick C 1 and 2 proteins (NPC1/2)-mediated cholesterol export from endolysosomes; or (ii) as a key protein involved in NPC1/2-independent cholesterol egress pathways^[12-14]^.

This study brings new insights into the specific molecular mechanisms by which CD63 influences the fate of cholesterol within cells, presenting potential therapeutic opportunities, particularly in conditions marked by dysregulated lipid metabolism, aberrant cholesterol homeostasis or pathologies associated with cholesterol accumulation, such as Niemann-Pick disease (NPC), neurodegenerative disorders, or atherosclerosis^[15]^. Kestecher and colleagues support this by demonstrating how altered CD63+ EV levels correlate with hypercholesterolemia and atherosclerosis in murine models, particularly when low-density lipoproteins (LDL) levels are elevated due to diet or genetic modifications. Their findings underscore the inverse correlation between CD63+ EVs and cholesterol levels, a relationship with potential implications for cardiovascular risk biomarkers^[16]^. In parallel, Bonora and colleagues elucidate how stem cell-derived EVs (SC-EVs) alter lipidomic profiles within target cells, suggesting that specific EV lipid cargo could be engineered for therapeutic purposes, particularly in stem cell applications to regulate lipid dysregulation. This work complements foundational findings on cholesterol-transporting proteins such as NPC1 and NPC2, further emphasizing the lipid regulatory potential of CD63 within the endosomal system^[17]^. Kestecher and colleagues also highlight that proprotein convertase subtilisin/kexin type 9 (PCSK9) knockout models show preserved cardiac function despite cholesterol elevation, suggesting that targeted modulation of EV levels could mitigate the cardiovascular consequences of lipid accumulation. Therapeutic interventions enhancing CD63 activity may improve lipid distribution within EVs, providing an innovative route for regulating cholesterol levels in hypercholesterolemic patients and potentially serving as adjunctive therapy with PCSK9 inhibitors^[16]^.

To summarize, Palmulli *et al*. brought the building block of promising outcomes that lipid-sorting proteins like CD63 could redefine approaches to managing lipid imbalances, offering novel therapeutic pathways for lipid-based pathological disorders. However, this CD63-mediated cholesterol egress pathway can be cell type-dependent^[10]^, and since lipid metabolism differs between humans and mice, the translation from mice experiments and human applications needs to be considered with care^[18]^. Moreover, studying the role of CD63 in cholesterol homeostasis would be of interest for cell type-specific events linked to hypercholesterolemia and cardiovascular diseases, such as those involving endothelial cells or monocyte-derived macrophages since EVs, and probably CD63^+^ EVs, play a role in these pathological contexts^[5,19-21]^. Lastly, and despite these discrepancies that remain to be elucidated, modulating CD63 activity or expression could, therefore, be of interest as a way to influence the cellular lipid composition to mitigate disease-associated lipid imbalances.

## DECLARATIONS

### Copyright

© The Author(s) 2025.
